# Association between triglyceride-glucose index and all-cause mortality in critically ill patients with acute myocardial infarction: analysis of the MIMIC-IV database

**DOI:** 10.3389/fendo.2025.1447053

**Published:** 2025-01-22

**Authors:** Xin Su, Yujing Zhou, Jie Chang, Xin Zhao, Haiyu Li, Haiqiang Sang

**Affiliations:** ^1^ Department of Cardiology, The First Affiliated Hospital of Zhengzhou University, Zhengzhou, China; ^2^ National Center for Neurological Disorders, Xuanwu Hospital, Capital Medical University, Beijing, China; ^3^ Department of Cardiology, Beijing Anzhen Hospital, Capital Medical University, Beijing, China

**Keywords:** triglyceride-glucose index, acute myocardial infarction, insulin resistance, all-cause mortality, MIMIC-IV database

## Abstract

**Background:**

Currently, the clinical evidence regarding the prognostic significance of the TyG index in acute myocardial infarction (AMI) patients remains unclear. Our research analyzed the correlation between the TyG index and the risk of mortality in patients with AMI, in order to evaluate the influence of the TyG index on the prognosis of this population.

**Methods:**

1205 ICU patients with AMI were analyzed in this retrospective cohort analysis, and the necessary data were obtained from the Medical Information Mart for Intensive Care IV (MIMIC-IV) database. The study conducted Kaplan-Meier analysis to compare all-cause mortality rates across four groups of patients. The study included logistic regression and Cox regression analysis to examine the correlation among the TyG index and the risk of in-hospital, 28-day, and 90-day mortality.

**Results:**

In our study, 176 (14.61%) patients experienced in-hospital deaths, 198 (16.43%) patients died within 28 days of follow-up, and 189 (23.98%) patients died within 90 days of follow-up. Logistic regression and Cox proportional hazard analyses revealed that the TyG index was an independent predictor of in-hospital, 28-day, and 90-day mortality (OR: 1.406, 95% CI 1.141-1.731, p = 0.001; HR: 1.364, 95% CI 1.118-1.665, p = 0.002; HR: 1.221, 95% CI 1.024-1.445, p = 0.026, respectively). The restricted cubic spline regression model showed that the risk of in-hospital, 28-day, and 90-day mortality increased linearly with increasing TyG index.

**Conclusions:**

The TyG index was significantly associated with an increased risk of mortality in AMI patients. Our findings suggested that the TyG index may be instrumental in identifying patients at high risk for adverse outcomes following AMI.

## Introduction

Despite significant advancements in the diagnosis and management of acute myocardial infarction (AMI), it is still the major cause of death on a global scale (1, 2). Although patients with AMI get treatment involving coronary revascularization, dual antiplatelet therapy, and aggressive lipid-lowering, some individuals, especially those with type 2 diabetic mellitus (T2DM), remain a significant likelihood of experiencing recurrent cardiovascular events (3). Moreover, AMI also imposes a substantial economic burden, particularly in nations with lower and moderate incomes (4). Coronary artery disease (CAD) significantly contributes to illness and death in diabetes (DM) patients, with diabetes-related cardiovascular disease expenditure estimated at $37.3 billion annually (5).

Insulin resistance (IR) is a defining characteristic of metabolic syndrome (MetS) (6), it is a major pathogenic mechanism of DM and a contributing factor to the progression of macrovascular complications in patients, and many studies have demonstrated the role of IR and disorders of glucolipid metabolism in the progression of CAD (7, 8). Methods for the assessment of IR include hypoglycaemic-hyperinsulinaemic clamp test, homeostatic model assessment of insulin resistance (HOMA-IR) insulin resistance index, but these methods are costly (9). In addition, researchers have found that TyG is measured by measuring fasting blood glucose (FBG) and triglycerides, correlates with the above methods in clinical practice and can be utilized as a specific and reliable predictor of IR (10).

IR predisposes individuals to metabolic disorders, including hyperglycemia and dyslipidemia, which are strongly associated with a poor prognosis in CAD. Some studies shown that TyG levels are valuable for prognostic prediction in CAD patients (11–13). Ryo et al. (14) found the higher the index, the worse the prognosis. As the TyG index increases, the danger about cardiovascular mortality or rehospitalization also increases (15). Nevertheless, there is currently insufficient clinical evidence to determine the validity of the TyG index in AMI patients. Our study explored the association between the TyG index and the prognosis of AMI patients admitted to the intensive care unit (ICU), to provide more meaningful information for clinical practice.

## Methods

### Data sources

Our study is a retrospective observational analysis that uses data from the publicly accessible Medical Information Mart for Intensive Care IV (MIMIC-IV) database (https://mimic.mit.edu). The MIMIC-IV researches stem from an organization between Beth Israel Deaconess Medical Centre (BIDMC) and the Massachusetts Institute of Technology (MIT). Data collected by Beth Israel Deaconess Medical Centre can be identified and transformed by researchers who, of course, have completed human research training and signed data use agreements. Meanwhile, the Beth Israel Deaconess Medical Centre Institutional Review Board has approved the waiver of informed consent and the sharing of research resources (16). In compliance with the applicable requirements, the author acquired a Collaborative Institutional Training Initiative (CITI) license and the requisite authorizations to make use of the MIMIC-IV database.

**Figure 1:**
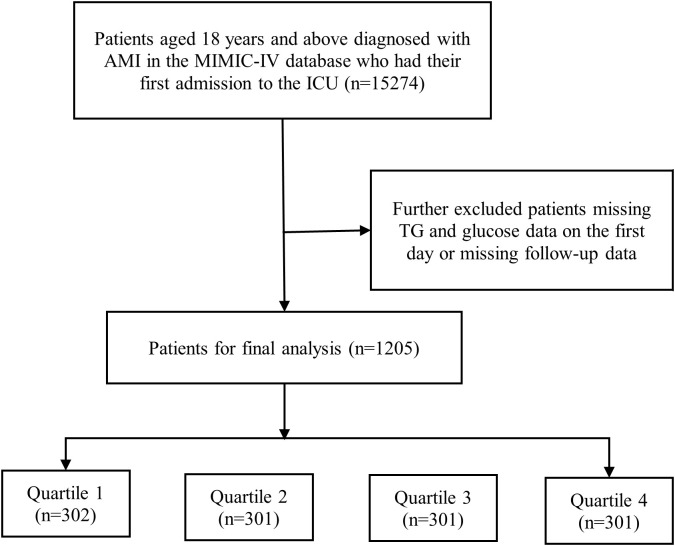
Flow chart of the study participants. AMI, acute myocardial infarction; MIMIC, Medical Information Mart for Intensive Care; TG, triglyceride.

Our study encompassed a total of 15,274 individuals who suffered from AMI and were hospitalized to the intensive care unit. All participants were 18 years of age or older in the research. **Figure 1** displays the flow chart. We exclusively analyzed the initial admission for patients with multiple admissions. In order ensure the precision and accuracy of the data, individuals who did not have AMI data available within 48 hours of being admitted to the ICU, had inadequate data on their glucose and triglyceride (TG) levels, or lacked follow-up data were excluded in our study. Ultimately, we established a final cohort of 1,205 patients, these patients were divided into four groups (Q1, Q2, Q3, Q4) based on the TyG index quartiles, and group Q1 was used as the reference group.

### Data collection

The baseline features of patients were extracted by collecting data using Structured Query Language (SQL) with PostgreSQL (version 16.2). The data included patient demographics (age, gender, body mass index (BMI)), Laboratory tests (white blood cell (WBC), platelet (PLT), red blood cell (RBC), total cholesterol (TC), TG, glucose, triglyceride glucose (TyG), partial thromboplastin time (PTT), prothrombin time (PT), international normalized ratio (INR), serum creatinine (Scr)), comorbidities (anemia, atrial fibrillation (AF), CAD, DM, heart failure, hyperglycemia, hypertension, transient ischemic attack (TIA), peripheral vascular disease (PVD), cerebrovascular disease, dementia, chronic pulmonary disease, renal disease), medication (statin, aspirin, clopidogrel), continuous renal replacement therapy (CRRT) and ventilation. The study’s follow-up period begins on the day of admission and ended upon the relevant endpoints occur.

To reduce potential bias, we excluded variables with missing values greater than 20%. Meanwhile, we carefully used multiple interpolation techniques to fill in the missing values for variables with less than 20% missing values, adopting a robust approach that maintains data consistency and reliability.

### Clinical outcomes

In our study, the primary outcome was in-hospital mortality, and secondary outcomes focused on 28-day and 90-day mortality.

### Statistical analysis

We used equation “ln [TG (mg/dl) × FBG (mg/dl)/2]” to calculate the TyG index.

For continuous variables, we used t-tests or analyses of variance (ANOVA) for statistical analyses and reported them as mean ± standard deviation. For categorical variables, analyses were performed using chi-square tests or corrected chi-square tests and expressed as numbers (proportions).

Logistic regression and Cox regression analyses were used to investigate the correlation between the TyG index and the risk of hospitalization, 28-day and 90-day mortality. In Model 1, we only included the TyG index without any additional adjustments. In Model 2, we modified the model to include gender, age, and BMI. Model 3 was adjusted for age, gender, BMI, RBC, WBC, PT, PTT, anemia, AF, CAD, heart failure, statin use, aspirin use, clopidogrel use, ventilation, TG, TC, and INR. This adjustment considered feature selection results and clinical experience.

We employed Kaplan-Meier survival analysis to assess the occurrence of outcome events in separate stratified groups, categorized by the TyG index. We next examined any disparities detected using the log-rank test. In addition, we analyzed potential non-linear relationships between the TyG index and in-hospital mortality, 28-day mortality and 90-day mortality using 3-node multivariate restricted cubic spline (RCS) regression. Subgroup analyses were performed, taking into account gender, age, BMI, heart failure, atrial fibrillation, DM, and hypertension. Subsequently, the p-value for interaction was assessed.

For our statistical analyses, we used SPSS (version 25.0, IBM), STATA (version 17.0), and R (version 4.1.3, Austria) to perform statistical analyses. Statistical significance was defined as a two-sided P value of less than 0.05.

## Results

In our retrospective analysis, we examined 1,205 patients diagnosed with AMI using strict criteria for inclusion and exclusion. Their average age was 66.95 ± 13.90 years and 791 (65.64%) were female. Additionally, our study revealed 176 (14.61%) in-hospital deaths, 198 (16.43%) deaths within 28 days of follow-up, and 189 (23.98%) deaths within 90 days of follow-up.

### Baseline characteristics

In **Table 1**, we analyzed the baseline characteristics of AMI patients by the TyG index quartiles. Our findings indicate that patients who have higher TyG indices typically exhibit characteristics such as younger age, greater BMI, and raised levels of WBC, TC, TG, and glucose compared to patients with lower TyG index. In individuals with an elevated TyG index, the risk of developing DM is significantly higher. Furthermore, our analysis showed patients with a higher TyG index were more likely to take aspirin and clopidogrel but less likely to receive statin. Moreover, patients with relatively high TyG had increased mortality relative to those with lower TyG.

**Table 1:** Characteristics and outcomes of participants according to the TyG index quartiles.

Variable	Total (n=1205)	Quartiles of TyG				P-value
		Q1 (n=302)	Q2 (n=301)	Q3 (n=301)	Q4 (n=301)	
Demographic						
Age, years	66.95 ± 13.90	69.95 ± 15.31	69.21 ± 13.16	65.69 ± 13.37	62.92 ± 12.47	<0.001
Female, n (%)	791 (65.64)	202 (66.89)	194 (64.45)	203 (67.44)	192 (63.79)	0.732
BMI, Kg/m^2^	29.30 ± 6.80	27.49 ± 5.86	28.68 ± 6.05	29.68 ± 7.25	31.36 ± 7.33	<0.001
Laboratory tests						
RBC, m/uL	3.95 ± 0.81	3.85 ± 0.79	3.97 ± 0.78	3.97 ± 0.82	3.40 ± 0.86	0.126
WBC, K/uL	12.65 ± 6.78	10.70 ± 4.87	12.21 ± 6.74	12.82 ± 6.22	14.87 ± 8.22	<0.001
PLT, K/uL	219.46 ± 96.31	213.63 ± 89.11	229.31 ± 98.43	218.16 ± 96.35	216.75 ± 100.71	0.208
TC, mg/dL	155.11 ± 51.91	147.09 ± 46.32	156.06 ± 53.88	154.96 ± 48.66	162.37 ± 57.19	0.031
TG, mg/dL	150.24 ± 110.22	75.87 ± 22.68	108.37 ± 28.54	150.08 ± 50.13	266.87 ± 154.73	<0.001
Glucose, mg/dL	162.71 ± 80.49	111.17 ± 26.54	138.98 ± 38.96	162.80 ± 61.64	238.07 ± 105.08	<0.001
TyG	9.14 ± 0.74	8.27 ± 0.31	8.86 ± 0.13	9.29 ± 0.15	10.14 ± 0.48	<0.001
PT, sec	14.79 ± 6.19	15.07 ± 6.64	14.67 ± 6.07	14.61 ± 5.85	14.82 ± 6.20	0.800
PTT, sec	43.05 ± 25.34	44.16 ± 28.76	42.22 ± 23.19	42.49 ± 22.51	43.34 ± 26.47	0.780
INR	1.35 ± 0.55	1.35 ± 0.54	1.35 ± 0.48	1.34 ± 0.54	1.37 ± 0.63	0.891
Scr, mg/dL	0.95 ± 0.67	0.91 ± 0.59	0.92 ± 0.71	0.96 ± 0.67	1.00 ± 0.68	0.301
Comorbidities						
Anemia, n (%)	220 (18.26)	54 (17.88)	55 (18.27)	57 (18.94)	54 (17.94)	0.986
AF, n (%)	390 (32.37)	109 (36.09)	92 (30.56)	97 (32.23)	92 (30.56)	0.422
CAD, n (%)	891 (73.94)	215 (71.19)	227 (75.42)	230 (76.41)	219 (72.76)	0.441
DM, n (%)	162 (13.44)	14 (4.64)	37 (12.29)	40 (13.29)	71 (23.59)	<0.001
Heart failure, n (%)	532 (44.15)	116 (38.41)	136 (45.18)	135 (44.85)	145 (48.17)	0.102
Hypercholesteremia, n (%)	68 (5.64)	20 (6.62)	22 (7.31)	13 (4.32)	13 (4.32)	0.251
Hypertension, n (%)	231 (19.17)	55 (18.21)	60 (19.93)	69 (22.92)	47 (15.61)	0.140
TIA, n (%)	92 (7.63)	26 (8.61)	23 (7.64)	22 (7.31)	21 (6.98)	0.888
PVD, n (%)	38 (3.15)	14 (4.64)	8 (2.66)	9 (3.00)	7 (2.33)	0.374
Cerebrovascular disease, n (%)	336 (27.88)	94 (31.13)	89 (29.57)	83 (27.57)	70 (23.26)	0.156
Dementia, n (%)	45 (3.73)	18 (5.96)	10 (3.32)	13 (4.32)	4 (1.33)	0.024
Chronic pulmonary disease, n (%)	286 (23.73)	79 (26.16)	71 (23.59)	68 (22.59)	68 (22.59)	0.701
Renal disease, n (%)	269 (22.32)	67 (22.19)	58 (19.27)	67 (22.26)	77 (25.58)	0.325
Medication						
Statin, n (%)	872 (72.37)	199 (65.89)	223 (74.09)	228 (75.75)	222 (73.75)	0.032
Aspirin, n (%)	893 (74.12)	212 (70.20)	215 (71.43)	234 (77.74)	232 (77.08)	0.072
Clopidogrel, n (%)	408 (33.86)	97 (32.12)	85 (28.24)	110 (36.54)	116 (38.54)	0.036
CRRT, n (%)	97 (8.05)	19 (6.29)	10 (3.32)	22 (7.31)	46 (15.28)	<0.001
Ventilation, n (%)	963 (79.92)	223 (73.84)	242 (80.40)	243 (80.73)	255 (84.72)	0.010
Outcomes						
In-hospital mortality	176 (14.61)	33 (10.93)	37 (12.29)	39 (12.96)	67 (22.26)	<0.001
28-day mortality	198 (16.43)	39 (12.91)	45 (14.95)	44 (14.62)	70 (23.26)	0.003
90-day mortality	289 (23.98)	64 (21.19)	69 (22.92)	66 (21.93)	90 (29.90)	0.047

BMI, body mass index; RBC, red blood cell; WBC, white blood cell; PLT, platelet; TC, total cholesterol; TG, triglyceride; TyG, triglyceride glucose; PT, prothrombin time; PTT, partial thromboplastin time; INR, international normalized ratio; Scr, serum creatinine; AF, atrial fibrillation; CAD, coronary artery disease; DM, diabetes; TIA, transient ischemic attack; PVD, peripheral vascular disease; CRRT, continuous renal replacement therapy.

### Association between TyG index and mortality

Through using multivariate logistics and COX regression analysis, it was determined that the TyG index is independently associated with an increased risk of in-hospital mortality (OR 1.406; 95% CI 1.141-1.731; P = 0.001), 28-day mortality (HR 1.364; 95% CI 1.118-1.665; P = 0.002), and 90-day mortality (HR 1.221; 95% CI 1.024-1.455; P = 0.026). The validity of these findings was additionally verified in both adjusted models 2 and 3. The OR for in-hospital mortality in the highest quartile of the TyG index was 3.091, with a 95% CI of 1.631-5.867. The HR for 28-day mortality was 3.359, with a 95% CI of 1.811-6.230. The HR for 90-day mortality was 2.431, with a 95% CI of 1.409-4.194. These results were obtained using the lowest quartile as a reference (**Table 2**).

**Table 2:** The association between TyG groups and in-hospital, 28-day mortality and 90-day mortality.

	TyG n=1205	Quartiles of TyG				P for trend
		Q1 (≤8.64) n=302	Q2 (8.64-9.06) n=301	Q3 (9.06-9.57) n=301	Q4 (≥9.57) n=301	
In-hospital mortality						
Events (%)	176 (14.6)	33 (10.90)	37 (12.3)	39 (13.0)	67 (22.3)	
Model 1						<0.001
OR	1.406	1.000	1.142	1.213	2.334	
P (95% CI)	0.001 (1.141-1.731)		0.601 (0.694-1.882)	0.443 (0.741-1.988)	<0.001 (1.485-3.668)	
Model 2						0.001
OR	1.798	1.000	1.272	1.562	3.159	
P (95% CI)	0.002 (1.238-2.611)		0.390 (0.735-2.200)	0.122 (0.887-2.748)	<0.001 (1.672-5.966)	
Model 3						0.001
OR	1.759	1.000	1.302	1.553	3.091	
P (95% CI)	0.003 (1.210-2.556)		0.348 (0.750-2.259)	0.128 (0.881-2.738)	0.001 (1.631-5.867)	
28-day mortality						
Events (%)	245 (20.3)	47 (15.6)	58 (19.3)	57 (18.9)	83 (27.6)	
Model 1						0.001
HR	1.364	1.000	1.185	1.155	2.044	
P (95% CI)	0.002 (1.118-1.665)		0.471 (0.747-1.882)	0.544 (0.726-1.836)	0.001 (1.330-3.140)	
Model 2						<0.001
HR	1.607	1.000	1.258	1.381	2.800	
P (95% CI)	<0.001 (1.296-1.992)		0.338 (0.787-2.011)	0.184 (0.858-2.223)	<0.001 (1.769-4.431)	
Model 3						<0.001
HR	2.019	1.000	1.443	1.682	3.359	
P (95% CI)	<0.001 (1.408-2.896)		0.160 (0.865-2.407)	0.059 (0.981-2.885)	<0.001 (1.811-6.230)	
90-day mortality						
Events (%)	304 (25.2)	66 (21.9)	77 (25.6)	68 (22.6)	93 (30.9)	
Model 1						0.023
HR	1.221	1.000	1.106	1.044	1.586	
P (95% CI)	0.026 (1.024-1.455)		0.608 (0.752-1.626)	0.826 (0.708-1.540)	0.015 (1.095-2.297)	
Model 2						<0.001
HR	1.455	1.000	1.178	1.268	2.218	
P (95% CI)	<0.001 (1.204-1.759)		0.415 (0.794-1.748)	0.247 (0.848-1.894)	<0.001 (1.490-3.300)	
Model 3						0.003
HR	1.751	1.000	1.276	1.433	2.431	
P (95% CI)	0.001 (1.270-2.415)		0.270 (0.827-1.968)	0.124 (0.907-2.264)	0.001 (1.409-4.194)	

Model 1 was unadjusted.

Model 2 was adjusted by Age, Gender, BMI.

Model 3 was adjusted by Age, Gender, BMI, RBC, WBC, PT, PTT, Anemia, AF, CAD, Heart Failure, Statin, Aspirin, Clopidogrel, Ventilation, TG, TC, INR.

OR, odds ratio; HR, hazard ratio; CI, confidence interval.

**Figure 2:**
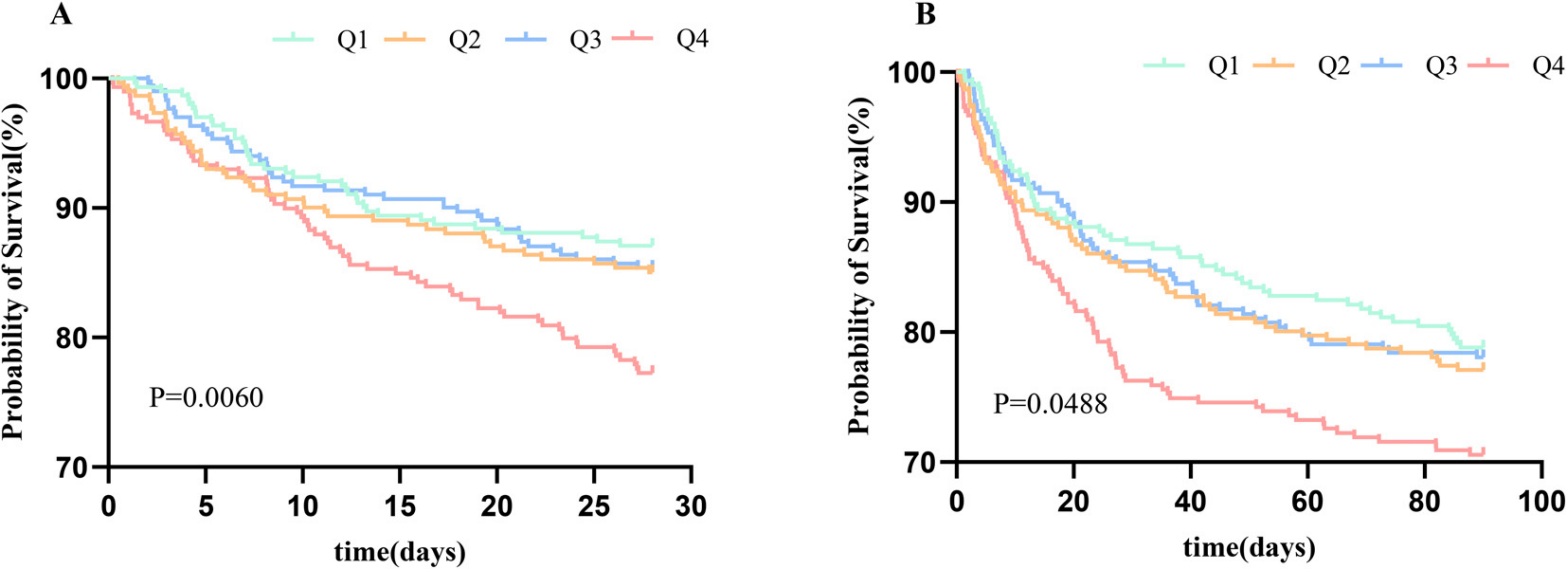
Kaplan–Meier survival analysis curve for 28-day mortality **(A)** and 90-day mortality **(B)**. TyG, triglyceride glucose index.

Kaplan-Meier survival analyses were used to assess the 28-day and 90-day mortality across TyG quartile groups. We observed statistical differences in the 28- and 90-day mortality rates in the quartile groups (log-rank p = 0.0060 and p = 0.0488, respectively) (**Figure 2**).

**Figure 3:**
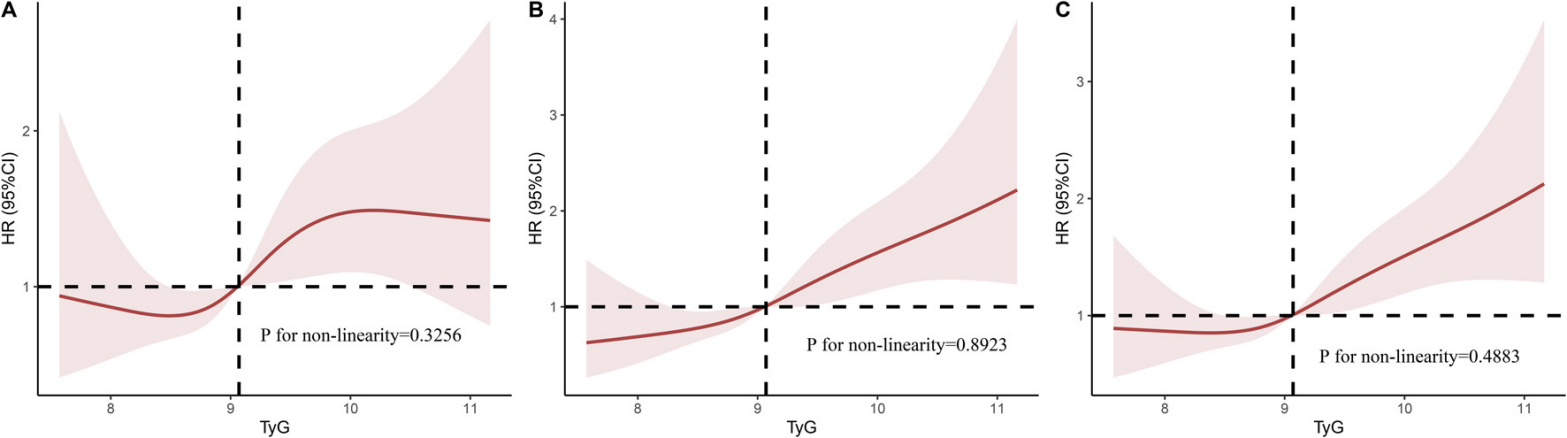
Multivariable RCS regression showed the nonlinear association between the TyG index and in-hospital mortality **(A)**, 28-day mortality **(B)**, and 90-day mortality **(C)** after adjusted by Age, Gender, BMI. TyG, triglyceride glucose; RCS, restricted cubic spline; OR, odds ratio; HR, hazard ratio.

**Figure 3** displays the RCS analysis results following the adjustment for age, gender, and BMI. We found that an elevated TyG index was linearly associated with higher in-hospital, 28-day, and 90-day mortality risk (P for nonlinear = 0.3256, 0.8293, and 0.4883, respectively).

### Subgroup analysis

**Figure 4:**
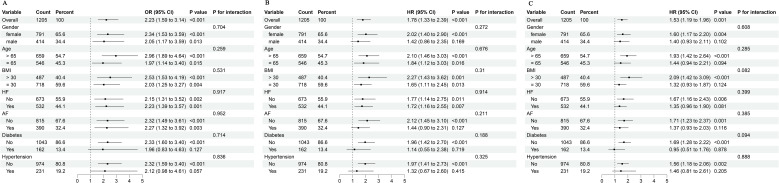
Subgroup analyses for the association of TyG index with in-hospital mortality **(A)**, 28-day mortality **(B)**, and 90-day mortality **(C)**. BMI, body mass index; HF, heart failure; AF, atrial fibrillation; TyG, triglyceride glucose; OR, odds ratio; HR, hazard ratio; CI, confidence interval.

We conducted stratified analyses based on gender, age, BMI, heart failure, atrial fibrillation, DM, and hypertension to confirm the link between TyG index and mortality. In **Figure 4**, our study demonstrated no significant interactions between baseline TyG index and stratification variables across subgroups (all p values for interaction > 0.05).

## Discussion

We studied the correlation between the TyG index and mortality in AMI patients. And the results of our research showed an important association between the TyG index and mortality in AMI patients. Particularly, this correlation persists even when adjusted for potential factors that might affect the results. Furthermore, the Kaplan-Meier analyses demonstrated that individuals with elevated TyG indices experienced worse clinical outcomes.

IR is strongly related to several disorders involving the metabolism of glycolipids, including hyperlipidemia, dyslipidemia and hypertension, mitral annular calcification, and cardiovascular prognosis (11, 12, 17). Prior researches demonstrated hypertension is the separate factor which may forecast platelet-dependent thrombosis. It worsens platelet-dependent thrombosis, raises the levels of adhesion molecules and leukocyte occlusion in capillaries, diminishes vasodilation, and hampers the availability of nitric oxide, which is crucial in preventing the formation of microthrombi after AMI (18, 19). Dysregulated insulin signaling impairs available nitric oxide, leading to vascular sclerosis (12). Additionally, IR has a significant impact on CVD through two distinct mechanisms: (1) atheromatous plaque formation. (2) ventricular hypertrophy and diastolic abnormalities (20). Both of these effects contribute to heart failure, impacting the prognosis of patients with CVD. Furthermore, Eddy et al. (21) found that IR may be the primary factor contributing to CAD. In addition, it has been demonstrated that IR is connected to damage in the arterial wall, including reduced vasodilatory function, heightened stiffness of arteries, and elevated coronary artery calcification, which have been identified as contributors to the development of future cardiovascular disease (22). Therefore, alleviation of IR or hyperinsulinemia is expected to be effective in reducing the likelihood of unfavorable outcomes in individuals with AMI. Of course, we emphasize that studies at the cellular and animal levels, as well as clinical trials which include large sample sizes and long-term follow-up are still needed to support this view. Further knowledge of the pathogenesis of IR and how it can be prevented or cured will have a profound impact on AMI.

The TyG index, compared to triglycerides, plasma atherogenicity index, and TG to HDL cholesterol ratio, is more effective in risk detection for cardiovascular events (23). Jin et al. suggest the TyG index might have superior predictive significance compared to the hemoglobin glycation index (HGI) in patients with stable CAD and DM (11). More importantly, the TyG index identifies IR reliably, not only due to the cost-effectiveness of serum TG and glucose level testing but also because it is fast and efficient (24).

Furthermore, the TyG index enables the timely identification of individuals with a heightened susceptibility toDM. After analyzing 2330 patients, Alessandra et al. revealed TyG index was positively correlated with the incidence in symptomatic CAD (25). A study conducted on 438 NSTE-ACS patients demonstrated a strong correlation between the TyG index was associated with the SYNTAX score and was an independent predictor for evaluating CAD severity (26). Hu et al. (27) discovered that patients with an rised TyG index exhibited a markedly increased susceptibility to cardiovascular events, regardless of whether they had DM or not. In CAD patients that received percutaneous coronary intervention (PCI), the TyG index can be a more reliable indicator than FBG or glycated hemoglobin. Additionally, Zhao et al. (28) suggested that the TyG index could predict a poor prognosis in AMI patients who have well-managed levels of LDL-C after PCI. Liu et al. (29) discovered that increased levels of the TyG index were suggestive of worsening IR and were non-linearly linked to all-cause and cardiovascular mortality. Within a study that included 2,830 participants, Zhao et al. (30) discovered a notable correlation between a higher TyG index and arterial stiffness and microvascular damage. In addition, a study of 888 participants with DM but without any prior cardiovascular disease (CVD) revealed that a higher TyG index was linked to an increased likelihood of CAD (31). However, there is an insufficient amount of current information about the correlation between the TyG index and AMI individuals. Our finding that TyG index is an independent factor for all-cause death in AMI patients adds to the existing body of research on the relationship between the TyG index and negative outcomes. Therefore, we appeal to clinicians to pay attention to patients’ glycemic management in IR indicators.

Our study demonstrates that the higher TyG index, the greater risk of negative outcomes in AMI patients, showing that the TyG index can be a beneficial indicator for assessing risk and guiding the clinical care of these patients. Liao et al. observed that the probability of death during hospitalization increased by 1.19 times for each 1-unit rise in the TyG index (32). This is in accordance with our findings. Our study found that when adjusted for confounders, each 1-unit rise in the TyG index was associated with a 1.019-fold rise in the probability of death within 28 days of follow-up. Therefore, clinical practitioners ought to actively manage risk factors associated with cardiovascular disease, including control of lipids and fasting blood glucose. Adopting systematic surveillance and intervention in individuals with high TyG index can significantly decrease the occurrence of negative outcomes in AMI patients.

Nevertheless, our study has limits. Firstly, our study was retrospective and observational, so the findings may indicate correlation more than causation. Therefore, future studies with multicenter prospective studies should be conducted. Secondly, due to the limitations of the MIMIC database, our study could not compare the TyG index with other IR measurement techniques and there was no way to confirm that all blood glucose and lipids were fasting results. Finally, the data was collected only from the United States, so our findings may not be fully applicable to ICU in other countries.

## Conclusions

Our findings provide further clinical evidence that the TyG index is valuable in the early identification of AMI patients with poor prognosis, thus helping to guide the clinical management of such patients. We also emphasize the importance of incorporating the TyG index into the daily routine of healthcare workers. Of course, further prospective studies are still needed to confirm our findings.

## Data Availability

The raw data supporting the conclusions of this article will be made available by the authors, without undue reservation.
